# Assessment of Mercury Concentration in Turtles (*Podocnemis unifilis*) in the Xingu River Basin, Brazil

**DOI:** 10.3390/ijerph15061185

**Published:** 2018-06-06

**Authors:** Marina Teófilo Pignati, Juarez Carlos Brito Pezzuti, Larissa Costa de Souza, Marcelo de Oliveira Lima, Wanderlei Antonio Pignati, Rosivaldo de Alcântara Mendes

**Affiliations:** 1 Programa de Pós-graduação em Zoologia, Universidade Federal do Pará e Museu Paraense Emílio Goeldi/CZO, Av. Perimetral, n.1, B. Guamá, Belém, PA 66075-750, Brazil; 2 Universidade Federal do Pará, Núcleo de Altos Estudos Amazônicos, Rua Augusto Corrêa, 01, Guamá, Belém, PA 66075-110, Brazil; juarez.pezzuti@gmail.com; 3 Instituto Evandro Chagas, Seção de Meio Ambiente, Laboratório de Toxicologia, Rodovia BR 316 km 07, Levilândia, Ananindeua, PA 67010-000, Brazil; laricsouza@gmail.com (L.C.d.S.); marcelolima@iec.pa.gov.br (M.d.O.L.); rosivaldomendes@iec.pa.gov.br (R.d.A.M.); 4 Instituto de Saúde Coletiva, Universidade Federal de Mato Grosso, Av. Fernando Corrêa da Costa, 2367, Boa Esperança, Cuiabá, MT CEP 78060-900, Brazil; pignatimt@gmail.com

**Keywords:** mercury, biomonitoring, aquatic toxicology, chelonian, Xingu

## Abstract

Many studies on mercury contamination in aquatic biota deal with the effect of consuming metal-contaminated organisms on human health. In this study, we examined the factors that cause mercury contamination in *Podocnemis unifilis* in the Xingu River Basin of Mato Grosso and Pará States, Brazil. We quantified by atomic absorption spectroscopy with cold vapor the total mercury (THg) content in the liver and muscle samples of 50 *Podocnemis unifilis* specimens collected from the basin. The liver and muscle samples contained 134.20 ± 119.30 ng g^−1^ THg and 24.86 ± 26.36 ng g^−1^ THg, respectively. Each chelonian or meal has, on average, 5.34× more Hg than the highest level established as acceptable. From the results it can be inferred that, given the weekly consumption of chelonians, the riverine and indigenous communities in the Xingu River Basin are at risk of chronic consumption of Hg in amounts beyond the acceptable limit. The potential high risk to the health of this population is evident; however, the risk classification needs to be further studied.

## 1. Introduction

Mercury (Hg) is a heavy metal found in both aquatic and terrestrial ecosystems [1,2]. Elevated Hg concentration is highly toxic, exerting adverse effects on the health of animals and humans [3,4]. Mercury poisoning in humans and vertebrate animals decreases their growth rate, blood cell viability, gonadal development, and even causes chronic convulsions or blindness [5,6,7,8,9].

Naturally occurring Hg in soil and vegetation biomass can be mobilized and its concentration can be elevated through anthropogenic activity. For instance, anthropogenic sources release approximately 1900–2900 Mg year^−1^ into the atmosphere, whereas the primary natural input is only 80–600 Mg year^−1^ [10].

The Amazonian region has the highest environmental concentration of natural Hg worldwide [11,12]. In Brazil, natural emission coupled with anthropogenic activities, such as mining, deforestation, and hydroelectric plants have considerably increased Hg release into the environment [2,13,14]. Moreover, other local environmental factors, such as aquatic pH and rainfall, influence the distribution of Hg [15,16].

Studies have demonstrated the extent of Hg accumulation in humans, animals, soil, and rivers [11,17,18,19,20,21]. Highly persistent, Hg is biomagnified along the food chain and is present in several aquatic trophic levels [19]. As ingesting contaminated food (especially seafood) is a major means of human exposure to Hg, studies have focused on Hg contamination in aquatic biota [22].

Given their long life span, aquatic turtles can accumulate toxic substances over extended periods and are thus important organisms for monitoring environmental contamination temporally [23,24,25]. Evidence from studies on turtles reveals that tissue Hg concentration is linked to variations in growth, size and tissue type [26,27,28].

Turtles represent an important source of food for Amazonian communities [29,30,31]. The aquatic turtle *Podocnemis unifilis* is widespread in the Xingu River Basin in the states of Mato Grosso to Pará [23,32]. Thus, there is a concern regarding how elevated Hg levels will affect the ecology of *Podocnemis unifilis* and how human consumption of *Podocnemis unifilis* might increase Hg exposure and contamination risk.

This study used *Podocnemis unifilis* as a biomonitor to investigate factors that affect the level of Hg contamination in five sites of the Xingu River Basin. We aimed to provide insights on a wide range of physical, ecological and anthropogenic factors that can influence environmental contamination and animal exposure risk. In addition to the abiotic variables, we examined whether physical size, sex and tissue type affect Hg accumulation in turtles.

## 2. Materials and Methods

### 2.1. Sampling Sites

**Figure 1:**
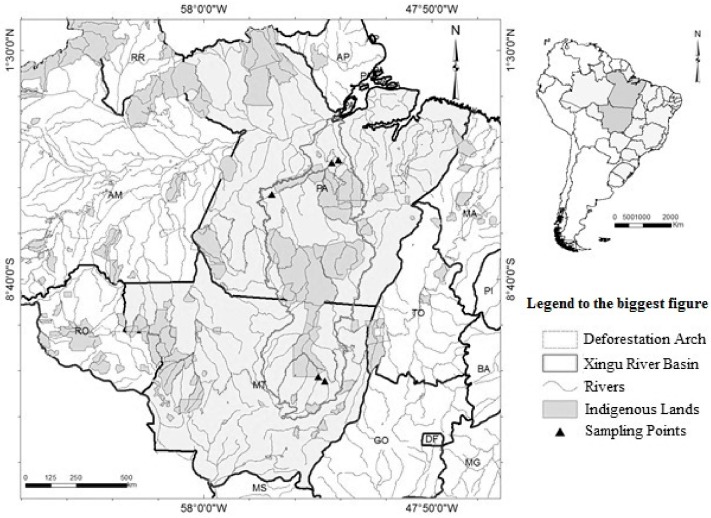
Map of the sampling localities in the Xingu River Basin of Mato Grosso and Pará States, Brazil (black triangles).

The Xingu River Basin spans 39 municipalities in the states of Mato Grosso and Pará (51 million hectares), covering 20 indigenous lands and 10 conservation areas. Located in an ecotone zone between the Cerrado–Amazônia biomes, the basin begins in Mato Grosso (Upper Xingu) with its mouth in Pará (Lower Xingu). The region has a population of about 610,000 (1.2 inhabitants/km^2^) that subsists mainly on cattle raising, monoculture, and wood extraction [33]. The study included five sampling points situated along the sources and tributaries of the rivers in the Xingu Basin (Figure 1, Table 1).

### 2.2. Animal Collection, Tissue Sampling, and Sample Preparation

Adult and juvenile turtles were captured during the dry season (October to December) of 2014, corresponding to the turtles’ reproductive season. Three methods were employed to capture adult and juvenile turtles. First, we used a hand-net consisting of a wooden rod attached to a small mesh sack via a ring. Second, we used fishing rods equipped with windlasses, and *Hoplias* spp. and *Gymnotus* spp. as bait. Finally, the divers manually caught specimens sighted on the water surface.

Ten *Podocnemis unifilis* individuals per locality were collected, yielding 50 specimens in total. The animals were sexed [32] and biometric data were obtained. The latter included straight carapace length (SCL) and plastron length measured with a pachymeter, as well as mass (g) determined using a scale. The collection of animals was authorized by the Chico Mendes Institute for Biodiversity Conservation No. 44743-1 and the Animal Ethics Committee for the Use of Animals at the Federal University of Pará No. 2661161216. The animals were euthanized in compliance with the guidelines for animal welfare. The muscle and hepatic tissue samples were obtained to avoid any metal contamination and stored in Falcon tubes at −20 °C until subsequent analyses.

### 2.3. Total Mercury (THg) Determination

The concentration of total Hg (THg) in the liver and muscles was analyzed using a previously published protocol [34]. Approximately 0.5 g of each sample was placed in a 50 mL volumetric flask, and 1 mL of deionized water, 2 mL of nitric acid plus perchloric acid solution (50:50), and 5 mL of concentrated sulfuric acid were added. The reaction mixture was subsequently heated for 30 min on a hot plate at 200 °C, cooled, and tested, with deionized water used as control. Furthermore, 5 mL of sample was subjected twice to cold vapor generation and atomic absorption spectrometry (CV–AAS). A calibration curve was then prepared by the same procedure [34], but altering the mass matrix through different volumes of standard Hg solution. The limits of detection (LD) and quantification (LQ) were 0.0001 and 0.0005 ng g^−1^, respectively.

### 2.4. Environmental Factors

Data on hotspots (sites with frequent fires) and deforestation (km^2^) in Mato Grosso and Pará during 2014 were obtained through the Fire Monitoring System of the PRODES project (Program for Calculating the Degree of Deforestation in the Amazon) [35,36]. Rainfall data (mm) for 2014 were obtained from the National Water Agency database [37]. For each body of water, the latitude was recorded, and pH at the time of sample collection was recorded using a pH field meter.

### 2.5. Statistical Analysis

Permutation tests for multivariate analysis of variance (PERMANOVA) were conducted to determine whether the THg concentration varied with the tissue type in *Podocnemis unifilis*. A similarity matrix based on Bray-Curtis Index was produced and Log(X+1) transformed for analysis, with 9999 permutations. A principal coordinates (*PCO*) analysis was performed to determine the tissue with the highest THg contamination. The analyses were conducted in PRIMER version 6 [38] with PERMANOVA+1 added [39].

The SCL, plastron length, and body weight correlated significantly (Pearson’s *r* ≥ 0.95, *p* < 0.05). Thus, only SCL was used in the subsequent statistical analyses. A covariance analysis (ANCOVA) was employed to examine the changes in the THg concentrations with body size and sex for each sampled tissue (sex and SCL of sampled turtles were entered as covariables). These analyses were conducted in Statistica version 10.0 [40].

Among the environmental variables, hotspot and deforestation were highly correlated (0.98), so were rainfall and latitude (0.91) (*p* < 0.05). Thus, only hotspot, rainfall, and pH were used in the multiple regression analysis on how the THg varied in each sampled tissue with environmental factors. These analyses were also conducted in Statistica.

A second PERMANOVA was performed for each sampled tissue to determine between-locality differences in the THg concentration. A Bray–Curtis-based similarity matrix was produced and Log(X+1) transformed with 9999 residual permutations in PRIMER version 6 with PERMANOVA+1. Significance was set at *p* < 0.05 for all analyses.

## 3. Results

### 3.1. Specimen Characterization

Table 2 shows the morphometrics of the sampled turtles.

### 3.2. THg Concentrations across Tissues

The mean THg concentration (and range) in the liver and muscle was 134.20 ± 119.39 (24.23–804.21 ng g^−1^) and 24.86 ± 26.36 ng g^−1^ (7.40–188.04 ng g^−1^), respectively.

**Figure 2:**
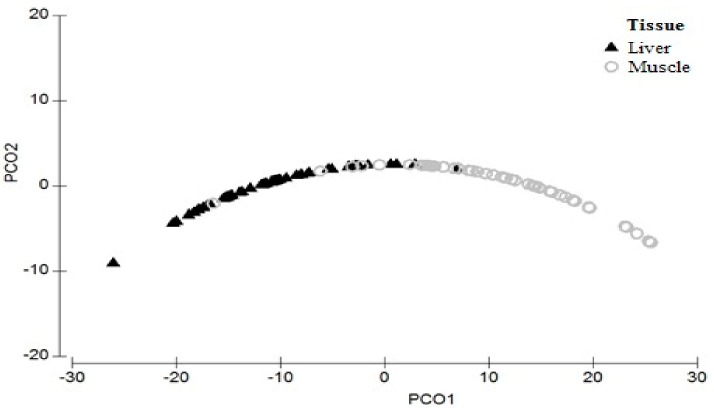
Principal coordinates analysis (PCO) of THg concentration (ng g^−1^) in the liver (black triangle) and muscle (grey circle) of *Podocnemis unifilis.*

The concentration of Hg was significantly higher in the liver than in muscle (pseudo-F_(1,98)_ = 165.06, *p* < 0.001). The PCO results identified two axes that accounted for 99.9% of the THg variation in both the tissues (PCO1 = 96.4% and PCO2 = 3.5%) (Figure 2).

### 3.3. Sex- and Size-Based Differences in THg Level

Female and male *Podocnemis unifilis* did not differ significantly with respect to concentrations of THg (ng g^−1^). The liver and muscle of male *Podocnemis unifilis* contained 136.48 and 24.70 ng g^−1^ THg, respectively, whereas that of female *Podocnemis unifilis* contained 138.04 and 25.77 ng g^−1^, respectively. Overall, neither the sex nor SCL significantly influenced the concentration of THg in the liver (ANCOVA, F_(1,46)_ = 0.351, *p* < 0.556) or muscle (F_(1,46)_ = 2.205, *p* < 0.144) of *Podocnemis unifilis*. Although no differences were found in the concentration of THg between the liver and muscle of *Podocnemis unifilis*, we found that the female with the highest SCL (33.2 cm) had a higher concentration of THg in the liver (253.96 ng g^−1^) and muscle (188.04 ng g^−1^).

### 3.4. Influence of Environmental Factors on THg Level

The environmental variables, including hotspots, rainfall, and pH, had no influence on the concentration of THg in the liver (multiple regression, *R*^2^ = 0.099, F_(3,46)_ = 1.6964, *p* < 0.180) or muscle (*R*^2^ = 0.569, F_(3,46)_ = 0.9258, *p* < 0.435).

### 3.5. Spatial Distribution of THg

The spatial analysis indicated that hepatic THg concentration was the highest (188.41 ng g^−1^) at PA3, located near the Belo Monte hydroelectric dam. Muscle THg concentration was the highest (36.35 ng g^−1^) at MT1 in the Sete de Setembro River, a source of the Xingu River. The turtle with the highest THg concentration (804.21 ng g^−1^) was also found in PA3. The lowest average hepatic THg concentration (77.31 ng g^−1^) was recorded at MT2 in the Culene River, also near the source of the Xingu River, whereas the lowest muscle THg concentration (15.89 ng g^−1^) was measured at PA1 in the Anfrísio River of Pará.

**Figure 3:**
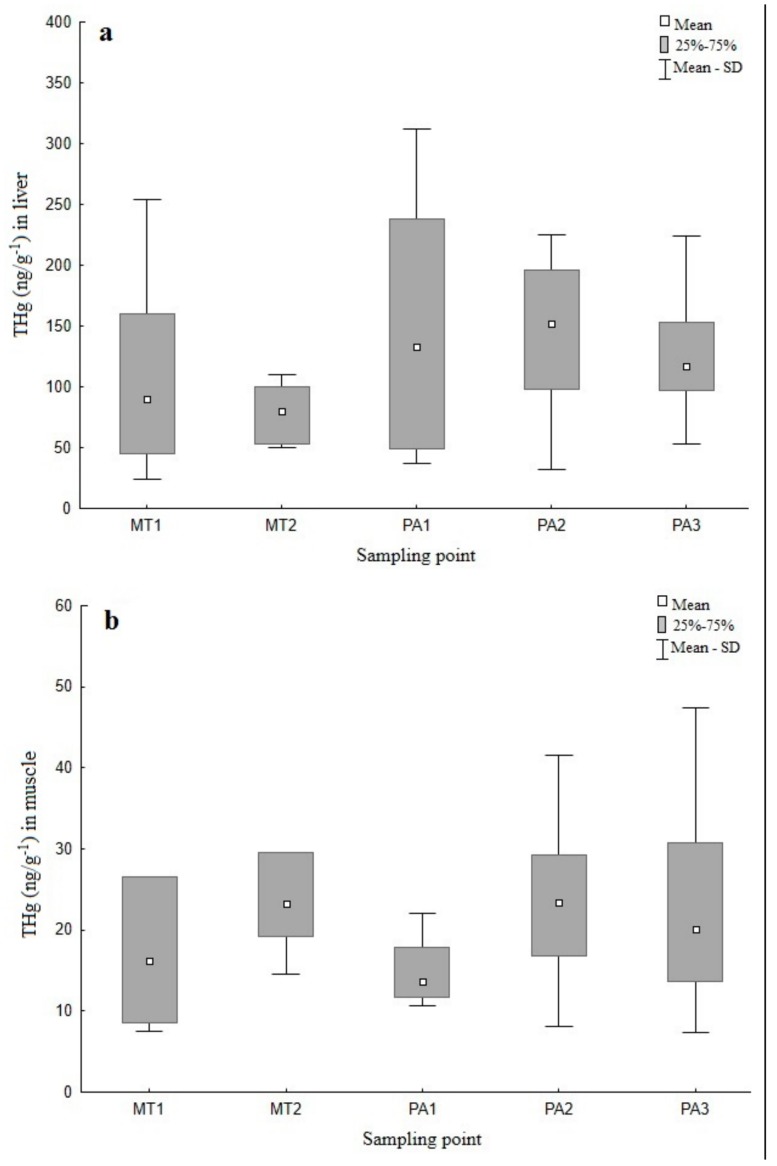
Total mercury concentration (THg) (ng g^−1^) in the liver (**a**) and muscle (**b**) of *Podocnemis unifilis* across sampling localities.

Overall, the concentration of THg did not vary significantly between the sampling localities (Figure 3a,b), either in the liver (PERMANOVA, pseudo-F_(4,45)_ = 1.4982, *p* < 0.2103) or muscle (pseudo-F_(4,45)_ = 1.1424, *p* < 0.3417).

## 4. Discussion

Mercury was detected in all tissue samples at varying concentrations. However, the concentrations observed were less than the levels reported in previous studies on turtle species, except those carried out in the Xingu Basin.

Souza-Araújo et al. [28] reported a concentration of 20 ng g^−1^ THg in the muscle samples of *Podocnemis unifilis* in the Xingu Basin, state of Pará. This is similar to the findings of the present study. However, several studies, including studies in the Amazon region, reported concentrations higher than that observed in the present study (Table 3); this can be accounted for by the fact that some of these studies were conducted with species at different trophic levels and in different geographic regions worldwide.

Since the 1960s, anthropogenic activities, such as mining, deforestation, and site burning, in northern Mato Grosso and Pará (Amazon region) have elevated the level of Hg emissions into the atmosphere. Therefore, Hg has gradually penetrated different habitats [44]. Even today, numerous illegal mining ventures continue to operate across the region, including indigenous lands and conservation zones of the Xingu River Basin in Pará. These activities, which cannot be mapped easily or effectively [33], lead to unchecked distribution of Hg throughout the basin, thus increasing the level of Hg that possibly contaminates aquatic organisms, such as *Podocnemis unifilis*.

The liver tends to accumulate high concentrations of toxic substances because it is an organ with high metabolic activity. Thus, it accumulates more metals than organs with low metabolic activity (such as muscle) [45]. The results of the present and previous studies indicate that the concentration of Hg is higher in the liver than in the muscle of several aquatic turtle species [20,28,41]. The reptilian liver is known to metabolize and store foreign chemical compounds (xenobiotics). These residues can accumulate to toxic levels if not excreted effectively [46]. Additionally, as detoxification systems in reptiles are less efficient than those in the endothermic organisms, the determination of potential toxicity must also consider how the reptiles metabolize xenobiotics [46].

### 4.1. Influence of Sex and Body Size on THg Level

Several studies have reported positive correlations between body size and Hg concentration in aquatic turtles, including *Chelydra serpentina* [24], *Chelodina parkeri*, *Heosemys spinosa, Leucocephalon yuwonoi*, *Malaclemys terrapin* [41], *Podocnemis erythrocephala* [20], and *Podocnemis unifilis* [43]. Among *Chelydra serpentina*, *Sternotherus odoratus*, *Chrysemys picta*, and *Pseudemys rubriventris*, the older and larger individuals tend to have proportionally higher Hg concentrations than the younger and smaller individuals [27]. This pattern probably occurred because Hg continually bioaccumulates over time or because the animals change their feeding habits as they increase in size. However, in the present study, we noted that although the morphometric measures can predict longevity, reptilian growth patterns are not always straightforward to interpret and should be treated with caution [47]. Several studies did not observe sex differences in total Hg concentration among aquatic turtles [21,27,28,41]. However, sex differences in the accumulation of contaminants are actually common in reptiles [26]. Such variations likely occur among reptiles because the females eliminate a portion of accumulated metals through egg laying. Other behavioral variations between the males and females might also explain differences in bioaccumulation. The female turtles also tend to be heavy, increasing the likelihood of organometal accumulation, thus altering the degree of exposure between sexes [48].

### 4.2. Influence of Environmental Factors on THg Level

Mercury contamination results from a combination of naturally occurring and anthropogenically produced Hg. In this study, we did not find a correlation between the THg concentration in *Podocnemis unifilis* and measured environmental variables or site locality. However, previous studies have suggested that the accumulation of Hg in the Amazonian animals reflects the bioavailability of metals in the environment [17].

The findings of this study are in line with those of previous studies [20,42], showing that the water pH is not correlated with the concentration of Hg in *P. erythrocephala* and *C. serpentina*, although environmental changes (including pH fluctuation) can alter mercury bioavailability. Methylation decreases with decrease in the water pH, therefore methylmercury is more likely to be present in areas with low pH, leading to its bioaccumulation throughout the food chain [15,16,49]. Clear rivers, such as the Xingu River and its tributary Iriri, are acidic (4.5–7.8), suggesting that Hg concentration in *Podocnemis unifilis* should also be high. However, as mentioned earlier, this was not the case in this study. The lack of significant variation in pH across the sampled water bodies might be a reason for this apparent contradiction.

The Amazon Basin is estimated to emit 6–8 tons of Hg per year due to burning forests [50]. As Hg does not mix with ash, precipitation causes the metal to exit soil as leachates that flow into bodies of water [51]. In the Xingu River and its tributary Iriri, a small amount of light material tends to be suspended in the water [49], and heavy rainfall benefits this process. Thus, we expected that burning forests and precipitation should influence Hg contamination in *Podocnemis unifilis*. However, we did not observe any evidence of this in this study. Our findings might reflect a generalized pattern of contamination affecting the Xingu Basin and possibly other sub-basins in the Amazon region. This overall trend is likely due to multiple anthropogenic activities that are also linked to naturally occurring Hg in the soil and ecosystem.

### 4.3. Spatial Distribution of THg

Our results revealed that the overall highest average THg concentration and also the turtle with the highest THg concentration in the liver were found at PA3, located near the Belo Monte hydroelectric dam. Thus, the proximity of individuals to this hydroelectric dam might have resulted in a high level of THg in turtles.

Hydroelectric dams are one of the anthropogenic features that can increase Hg contamination in *Podocnemis unifilis* and elevate Hg exposure risk among the communities in the Xingu consuming these turtles. The dams increase methylmercury production by slowing down water flow upstream, thus allowing Hg accumulation and mixing with the river-bottom sediment. Eventually, Hg and sediment tend to separate in the dam reservoir. Simultaneously, anoxic conditions in the dams cause methanogenic bacteria to proliferate, increasing methylmercury production and elevating contamination risk throughout the food chain [52]. Currently, the operational dams in the Amazon Basin include the Paranatinga II, Culuene, and Ronuro hydroelectric dams in Mato Grosso, as well as the Belo Monte hydroelectric dam in Pará. These structures potentially spread Hg contamination in aquatic life in the Xingu River Basin and hence in the inhabitants of the surrounding regions.

### 4.4. Evaluation of Risk to Human Consumers of Turtles

Food consumption is the main source of human exposure to contaminants, representing a fundamental component of health risk assessment [53,54,55]. The exposure to chemical substances in the diet can be chronic or acute, with chronic exposure characterized by the ingestion of small quantities of a substance over a long period and acute exposure by the ingestion of large quantities during an interval of up to 24 h [56].

The chelonians of the genus *Podocnemis* represent an important source of diet of the Amazonian population, with the consumption of one individual of these species per meal/week/person in the Parque Nacional do Jaú, in the state of Amazonas, as well as in the Bolivian Amazon [29,57]. With the communities in the Amazon still consuming chelonians [29], it is important to establish safe consumption limits of this protein resource.

To analyze the health risk, Green et al. [41] was referenced regarding the acceptable limit of Hg in chelonians because these authors adapted the limits set by United States Environmental Protection Agency (EPA) and the Food and Drug Administration (FDA). They have established an acceptable daily intake of Hg in fish. Based on their study by Green et al. [41], which also established the consumption limit of 1900 ppb Hg for a meal of 277 g of chelonian, i.e., 6.86 ng of mercury per gram of edible part of the turtle (muscle and liver), we evaluated the potential risk of Hg by the consumption of the chelonians sampled in this study.

According to Rodrigues et al. [58] and Luz et al. [59], the body weight of *Podocnemis* spp. is equivalent to 24.1% of muscle tissue and 2.90% of liver based on the total body weight. In the present study, the average body weight of the turtles was 1.717 g; thus, the average weight of muscle and liver of each chelonian was 414 g and 50 g, respectively. The results showed that the average amount of Hg in 1 g of muscle is 24.86 ng and in 1 g of liver is 134.20 ng of Hg. Thus, it was found that a chelonian on average contains 17,002 ng Hg or 36.62 ng g^−1^ Hg in its edible parts (muscle and liver). Consequently, each chelonian or meal consisted of, on average, 5.34× more Hg than the highest level established for Green et al. [41] by the EPA.

From the results it can be inferred that, given their weekly consumption of chelonians, the riverine and indigenous communities in the Xingu River Basin are at risk of chronic uptake of mercury in amounts beyond the acceptable limit. Thus, the potential high risk of Hg to the health of this population is evident; however, the risk classification needs further studies.

Thus, turtle meat is frequently consumed by the riverside and indigenous communities in the Xingu River Basin, indicating that these communities are at a risk of suffering health problems related to Hg exposure.

## 5. Conclusions

All turtle tissue samples exhibited detectable concentrations of THg, with the liver containing THg levels significantly higher than that of muscles. Neither sex nor body size significantly influenced the concentration of THg in turtles. Similarly, the measured environmental factors did not affect the concentration of THg. This species is becoming an important biomonitor of environmental quality and Hg exposure risk. Furthermore, *Podocnemis unifilis* is a popular source of food for the native communities in the Xingu River Basin. Each chelonian or meal has, on average, 5.34× more Hg than the highest level established as acceptable. Given the weekly consumption of chelonians, the riverine and indigenous communities in the Xingu River Basin are at risk of chronic consumption of mercury in amounts beyond the acceptable limit. We strongly recommend further studies to empirically determine the level of Hg in river sediment, air, soil, and riverside human inhabitants.

## Figures and Tables

**Table 1:** Sampling location in the Xingu River Basin of Mato Grosso and Pará States, Brazil.

			Geographic Coordinates	
Code	Municipality	Description	Latitude	Longitude
MT1	Canarana	Sete de Setembro River	13°10′57.4″ S	52°34′35.7″ W
MT2	Gaúcha do Norte	Culuene River	12°59′06.4″ S	52°52′42.8″ W
PA1	Altamira	Anfrísio River	04°53′17.7″ S	54°55′57.2″ W
PA2	Altamira	Belo Monte Hydroelectric Dam	03°29′10.6″ S	52°15′50.2″ W
PA3	Altamira	Belo Monte Hydroelectric Dam	03°22′16.6″ S	51°57′51.3″ W

**Table 2:** Distribution of sampled *Podocnemis unifilis* individuals by sex and body size.

	*N*	SCL (cm)	Weight (kg)
**M**	28	24.4 ± 4.0 (17.8–27.8)	1.525 ± 0.788 (0.600–2.100)
**F**	22	25.6 ± 4.1 (22.8–33.2)	1.758 ± 0.814 (1.200–3.800)
**Total**	50	25.5 ± 4.0 (17.8–33.2)	1.717 ± 0.790 (0.600–3.800)

M = male; F = female; average values ± standard deviation (minimum-maximum); SCL (straight carapace length).

**Table 3:** Published data on mercury (Hg) concentration in turtles.

Species	Concentration of Hg (ng g^−1^)		Reference
	Liver	Muscle	
*Chelodina parkeri*	593	329	[41]
*Chelus fimbriata*	-	432	[21]
*Chelydra serpentina*	50–500	-	[24]
*Chelydra serpentina*	-	48.1	[42]
*Heosemys spinosa*	137.9	10	[41]
*Leucocephalon yuwonoi*	78	4	[41]
*Malaclemys terrapin*	149.3	54	[41]
*Peltocephalus dumerilianus*	-	106	[21]
*Podocnemis erythrocephala*	470	33	[20]
*Podocnemis erythrocephala*	-	33	[21]
*Podocnemis expansa*	-	62	[21]
*Podocnemis expansa*	-	1	[43]
*Podocnemis sextuberculata*	-	61	[21]
*Podocnemis unifilis*	-	34	[21]
*Podocnemis unifilis*	-	1	[43]
*Podocnemis unifilis*	-	20	[28]
*Podocnemis unifilis*	134.20	24.86	Present study
